# Systematic Identification and Functional Validation of CASP10 as a DNA‐Damage‐Responsive Driver of Endothelial Pyroptosis in Atherosclerosis

**DOI:** 10.1111/jcmm.71060

**Published:** 2026-02-17

**Authors:** Xiangrong Meng, Kejian Zhang, Yu Xi, Zhuozhong Wang, Xinyu Zhu, Wenjing Zhang

**Affiliations:** ^1^ Department of Laboratory Diagnosis The Second Affiliated Hospital of Harbin Medical University Harbin Heilongjiang China; ^2^ Department of Orthopedic Surgery The Second Affiliated Hospital of Harbin Medical University Harbin China; ^3^ The Key Laboratory of Myocardial Ischemia Harbin Medical University, Ministry of Education Harbin Heilongjiang China; ^4^ College of Bioinformatics Science and Technology Harbin Medical University Harbin Heilongjiang China

## Abstract

Atherosclerosis (AS) is a chronic inflammatory disease driven by endothelial dysfunction and plaque instability. The DNA damage response (DDR) has been implicated in endothelial cell fate; its precise role in AS remains unclear. This study aims to identify DDR‐related biomarkers associated with AS and elucidate their mechanisms in endothelial pyroptosis. Our analysis identified 66 DDR‐related genes, which achieved over 80% accuracy in discriminating early from advanced lesions and hemorrhagic from non‐hemorrhagic plaques in external datasets, and reached 100% accuracy in differentiating plaque stability. CASP10 emerged as a top diagnostic biomarker (AUC = 0.991) compared to other DDR genes. Single‐cell analysis confirmed elevated CASP10 expression in endothelial cells of AS plaques. Functional experiments revealed that CASP10 is both necessary and sufficient for ox‐LDL‐induced DNA damage and pyroptosis in HUVECs. CASP10 overexpression exacerbated γH_2_AX accumulation, NLRP3 expression, GSDMD‐N cleavage and IL‐1β release, while CASP10 knockdown attenuated these effects. Overall, CASP10 serves as a reliable biomarker for identifying unstable plaques and functions as a pivotal mediator linking DNA damage to endothelial pyroptosis. Targeting CASP10 may represent a novel therapeutic strategy to reduce endothelial cell death and stabilise atherosclerotic plaques.

## 1 Introduction

Atherosclerosis (AS) is a chronic inflammatory disease characterised by lipid accumulation and endothelial dysfunction, serving as the primary pathophysiological basis for ischemic heart disease [1]. The principal complication of ischemic heart disease is myocardial infarction, which results from the abrupt occlusion of the coronary artery supplying myocardium and is a leading cause of mortality worldwide [2]. The primary causes of coronary artery occlusion are plaque rupture (occurring in 75% of cases) and thrombosis, both indicative of advanced atherosclerosis [3, 4]. Despite extensive investigation, the precise molecular mechanisms underlying the progression of atherosclerotic plaques remain elusive. Endothelial cells, acting as the interface between the vascular system and underlying tissues, play a pivotal role in maintaining vascular homeostasis by regulating vascular tone and exerting anti‐thrombotic and anti‐inflammatory effects [5]. Endothelial dysfunction, a key factor in plaque formation, progression and instability, triggers vascular inflammation [6]. However, the mechanisms underlying endothelial dysfunction are complex and require further elucidation. Therefore, there is a continuous need to identify novel biomarkers to provide a basis for risk stratification and the development of new therapeutic strategies for patients with atherosclerotic plaques.

Double‐strand breaks, oxidative base adducts and telomere attrition converge on the canonical DNA‐damage response (DDR) network, which is primarily orchestrated by the ataxia‐telangiectasia mutated (ATM)/ataxia telangiectasia and Rad3‐related (ATR)‐p53 axis and poly (ADP‐ribose) polymerase (PARP) [7, 8]. The recruitment of either ATM or ATR to the site of damage initiates the local phosphorylation of histone H_2_AX, a pivotal event in DDR initiation [9, 10]. This evolutionarily conserved pathway can sense DNA damage and transmit this information throughout the cell by activating a signalling cascade known as the DDR. This response has two different but coordinated functions: it can prevent or inhibit the replication and distribution of damaged DNA to daughter cells, thereby preventing the spread of genetic errors; it also coordinates efforts within the cell to repair DNA damage and maintain genomic integrity [8]. If DNA damage in proliferating cells can be repaired promptly and correctly, the cells will quickly return to normal proliferation. In contrast, when DNA damage is particularly severe, the cell may initiate the process of cellular senescence, which is a natural irreversible cell cycle arrest triggered by the DNA repair signalling pathway. However, there is also a possible outcome where the cell may undergo programmed cell death, mainly through apoptosis, which is a form of cell suicide and can remove the damaged cell from the cell population [11]. This process is crucial for preventing the propagation of cells with irreparable DNA damage and maintaining tissue homeostasis. Mounting evidence now positions persistent endothelial DNA damage not as a passive correlate of plaque burden, but as an early, causal trigger that accelerates atherogenesis by driving maladaptive inflammatory responses and structural destabilization [12, 13]. While endothelial DNA damage has been causally implicated in the initiation and progression of atherosclerotic plaques, the biomarkers that reflect genotoxic stress‐driven pro‐inflammatory and plaque‐destabilising responses remain largely uncharted.

Pyroptosis is a caspase‐1/4/5/11‐dependent, highly inflammatory and extensively propagated form of programmed lytic cell death [14, 15]. Upon activation, NLRP3 polymerises into a macromolecular scaffold that recruits the adaptor ASC (apoptosis‐associated speck‐like protein containing a CARD) and pro‐caspase‐1 [16]. The N‐terminal pyrin domain of NLRP3 drives proximity‐induced auto‐proteolysis of pro‐caspase‐1, yielding the catalytically active p20/p10 tetramer. Activated caspase‐1 then cleaves gasdermin‐D to liberate the pore‐forming N‐terminal fragment (GSDMD‐N) and simultaneously converts pro‐interleukin‐1β into mature IL‐1β. Pore insertion and membrane rupture release GSDMD‐N and IL‐1β into the extracellular space, amplifying cytokine circuits, recruiting macrophages and expanding the necrotic core [17]. We previously demonstrated that ox‐LDL triggers robust endothelial pyroptosis and accelerates atherosclerotic plaque formation, whereas independent studies have concurrently reported pronounced DNA‐damage foci in ox‐LDL‐challenged HUVECs [18, 19, 20]. Yet, the specific DDR effector genes that mechanistically bridge genotoxic stress to endothelial pyroptosis in atherosclerosis remain largely elusive.

**FIGURE 1:**
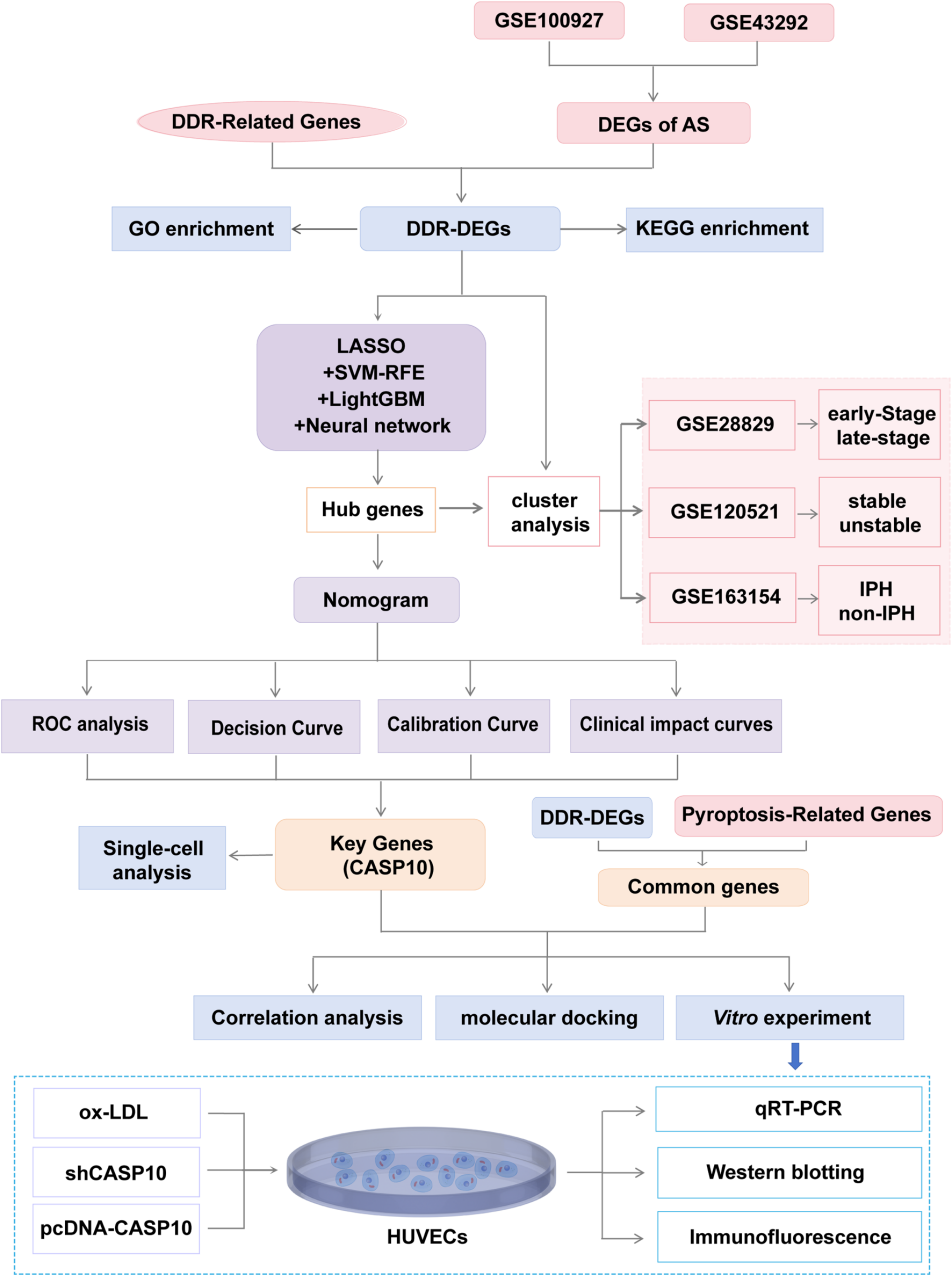
Study flow chart.

Conventional DDR proxies—p53, γH_2_AX and PARP1—are insufficient to decode the pleiotropic inflammatory landscape of human atherosclerotic endothelium. Exploiting high‐resolution transcriptomes, we interrogated the entire DDR module for pyroptosis‐regulating genes. By integrating bulk RNA‐seq from carotid plaques with endothelial single‐cell RNA‐seq data, we deployed an ensemble machine‐learning framework to derive a genome‐wide DDR signature, from which caspase‐10 (CASP10) surfaced as the dominant driver, effectively distinguishing the nature of atherosclerotic plaques. CASP10 expression was markedly upregulated in ox‐LDL‐stimulated endothelial cells and was necessary to amplify DNA damage (γH_2_AX) and trigger pyroptosis. ShRNA‐mediated knockdown of CASP10 markedly attenuated DNA‐damage‐induced pyroptosis in vitro (Figure 1). Collectively, our data establish CASP10 as a data‐defined therapeutic node for targeting the DDR‐pyroptosis axis in AS.

## 2 Materials and Methods

### 2.1 Data Acquisition

Six atherosclerotic microarray datasets (GSE100927, GSE43292, GSE28829, GSE120521, GSE163154 and GSE159677) were retrieved from the National Center for Biotechnology Information (NCBI) Gene Expression Omnibus (GEO) database (https://www.ncbi.nlm.nih.gov/geo/). All datasets were log‐normalised using a standard logarithmic transformation method. Specifically, GSE100927 was designated as the training set. GSE43292 was utilised as the validation set to evaluate the robustness and accuracy of the model constructed from the training set. The remaining datasets, GSE28829, GSE120521 and GSE163154, which contains various types of atherosclerotic plaques, were used to further validate the findings across different plaque types. Additionally, the single‐cell RNA‐sequencing (scRNA‐seq) dataset GSE159677 was incorporated to delineate the cell populations expressing key biomarkers in atherosclerosis. This dataset comprises carotid atherosclerotic plaques and patient‐matched proximal adjacent portions collected from three patients. Detailed information on the datasets, including the microarray platform, sample groups and numbers, is provided in File S1.

### 2.2 Identification of DDR Related Differentially Expressional Genes

The raw data from the GEO were normalised using the ‘NormalizeBetweenArray’ R package. The ‘limma’ package was utilised to perform differential analysis on the atherosclerotic datasets. Genes with differential expression (DEGs) were identified using the criteria of *p* < 0.05 and |log_2_FC| > 0.5. Volcano plots were generated using the ‘ggplot2’ package to visualise DEGs. We identified 4529 genes associated with DNA damage responses (DDR) from the GeneCards database (https://www.genecards.org/). These genes were selected based on their correlation scores exceeding the average and being protein coding. Venn diagrams were used to identify overlapping DNA damage response (DDR)‐DEGs between GSE100927, GSE43292 and DDR genes.

### 2.3 Functional Enrichment Analysis

Functional enrichment analysis was performed for the overlapping DDR‐DEGs using Gene Ontology (GO) terms, Kyoto Encyclopedia of Genes and Genomes (KEGG), Reactome and Wikipathways. The analysis was conducted using three methods: ‘clusterProfiler’ R package, SangerBox (http://sangerbox.com/) and Metascape (http://metascape.org/). To ensure robust statistical interpretation, the Benjamini–Hochberg false discovery rate (FDR) correction was applied for multiple testing, with a significance threshold set at *p* < 0.05.

### 2.4 Consensus Clustering Analysis

Patients with different subtypes of atherosclerosis (GSE28829, GSE120521 and GSE163154) were classified using the ‘ConsensusClusterPlus’ package in R based on the expression patterns of DDR‐DEGs. To identify the optimal grouping, the best combination was determined by utilising the consensus matrix plot, consensus cumulative distribution function (CDF) plot, and relative alterations in the area under the CDF curve. To account for multiple testing, the Benjamini–Hochberg false discovery rate (FDR) correction was applied, with a significance threshold set at *p* < 0.05.

### 2.5 Machine Learning‐Identified Hub Genes

We applied four machine learning approaches to identify DNA damage‐related signatures in atherosclerosis: LASSO regression using the ‘glmnet’ package, Support Vector Machine with Recursive Feature Elimination (SVM‐RFE) using the ‘e1071’ and ‘caret’ packages. LightGBM using the ‘lightgbm’ package and Neural Networks (NNET) using the ‘neuralnet’ package. Overlapping hub genes were identified among the four classification models using a venn diagram. To quantify the diagnostic value of the machine‐learning‐derived gene signature for atherosclerosis, we constructed a nomogram with the ‘rms’ package in R via binary logistic regression. The diagnostic performance was assessed using ROC curves, area under the curve (AUC), calibration curves, decision curves analysis and clinical impact curves were calculated to assess diagnostic performance.

### 2.6 Single‐Cell RNA Sequencing Data Analysis

The barcodes data, gene features data and gene count matrix data of GSE159677 from the GEO database were imported into R and analysed using the ‘Seurat’ package. Quality control was conducted by filtering out cells that satisfied the following criteria: the number of genes in each cell was in the range of 200 to 2500, and the content of mitochondrial RNA in each cell was < 5%. The data were normalised using the NormalizeData function. The combined object underwent principal component analysis (PCA) and Uniform Manifold Approximation and Projection (UMAP) analysis. A total of 35,784 filtered cells were selected for further analysis. The top 2000 variably expressed genes were determined using the ‘FindVariableFeatures’ function of the ‘Seurat’ package. The ‘FindClusters’ function was utilised to classify the cells into distinct clusters. Cell subsets were annotated using key marker genes through the ‘RenameIdents’ function. A comprehensive list of these markers is supplied in File S2.

### 2.7 Correlation Analysis

We identified 52 known pyroptosis genes from our previous studies [21]. The overlap between these pyroptosis genes and AS‐associated DEGs was determined using Venn analysis, resulting in a set of common genes. The correlation of CASP10 with these pyroptosis‐related genes was assessed using Sangerbox (http://sangerbox.com/home.html) and GEPIA (http://gepia.cancer‐pku.cn/).

### 2.8 Molecular Docking

The 3‐D structure of human CASP10 was obtained by retrieving its canonical FASTA sequence from UniProt (https://www.uniprot.org) and subsequently modelling it with AlphaFold2. The experimentally resolved structures of interleukin‐1β (IL1B; PDB ID: 1IOB) and the NOD‐like receptor family pyrin domain‐containing 3 (NLRP3; PDB ID: 3QF2) were downloaded from the RCSB Protein Data Bank (https://www.rcsb.org). Protein–protein docking between CASP10 and IL1B/NLRP3 was performed using the ZDOCK server (https://zdock.wenglab.org/) under default parameters. The resulting complexes were visually inspected and analysed with PyMOL (Schrödinger LLC) to assess the binding interfaces and interaction patterns.

### 2.9 Cell Acquisition and Cell Culture

Primary human umbilical vein endothelial cells (HUVECs) were isolated by enzymatic perfusion of umbilical cords obtained from the Second Affiliated Hospital of Harbin Medical University (Ethics Committee approval KY2022‐312). HUVECs were isolated from neonatal umbilical cords. The cords were rinsed three times with sterile phosphate‐buffered saline (PBS) to remove residual blood clots, followed by clamping the distal end with a hemostat. The cords were then incubated at room temperature in 0.1% type II collagenase (Biosharp, Anhui, China) for 30 min. Enzymatic digestion was terminated by adding RPMI‐1640 medium (Gibco, Thermo Fisher Scientific, USA) containing 10% fetal bovine serum (FBS) and 1% penicillin–streptomycin, which also served to rinse the vein. All effluent fluid was collected, centrifuged at 1000 rpm for 5 min, and the cell pellet was washed three times with sterile PBS. The cells were resuspended in complete endothelial cell medium (ECM, ScienCell Research Laboratories, USA) containing 5% FBS, 1% endothelial cell growth supplement (ECGS), and 1% penicillin–streptomycin (P/S) and transferred to a culture flask. Cells were maintained in a humidified incubator at 37°C with 5% CO_2_, and only HUVECs between passages 3 and 8 were used for experiments. To determine optimal conditions for CASP10 induction, HUVECs were treated with oxidised low‐density lipoprotein (ox‐LDL; Yiyuan Biotechnology, Guangzhou, China) at concentrations of 0, 25, 50 or 100 μg/mL for 24 h. A time‐course experiment was also conducted with 100 μg/mL ox‐LDL for 0, 12 or 24 h. PBS‐treated and lipopolysaccharide (LPS, 1 μg/mL; Solarbio)‐treated cells served as negative and positive inflammatory controls, respectively.

### 2.10 Gene Intervention

For in vitro experiments, we employed lentiviral transduction and plasmid‐based approaches to modulate gene expression. At 50% confluence, endothelial cells were transduced with lentivirus expressing CASP10 shRNA (General Biol, Anhui, China) at a multiplicity of infection (MOI) of 10 for 24 h. After medium replacement, 2 μg/mL puromycin was added and cells were monitored daily until > 95% puromycin‐resistant cells were observed by microscopy. For pcDNA3.1‐CASP10 plasmid (genepharma, Shanghai, China) transfection, when cells in 24‐well plates reached 70% confluence, Lipofectamine 3000 (Invitrogen, Carlsbad, USA) and the plasmid were separately diluted in Opti‐MEM (Thermo Fisher Scientific, Waltham, USA). The diluted solutions were incubated at room temperature for 5 min, combined, mixed gently, and allowed to complex for 20 min. The transfection complexes were added to the wells and incubated for 24 h. The pcDNA3.1‐CASP10 constructs are detailed in the File S3. After transfection, cells were cultured or stimulated with ox‐LDL for an additional 24 h before proceeding to the desired assays.

### 2.11 Quantitative Real‐Time PCR (qRT‐PCR)

Total RNA was isolated from HUVECs using TRIzol reagent (Thermo Fisher Scientific, Carlsbad, USA), and then reverse‐transcribed into cDNA with PrimeScript RT Master Mix (SEVEN Biotech, Beijing, China) for mRNA. qRT‐PCR was performed on a LightCycler 480 instrument (Roche, Basel, Switzerland) using 2× SYBR Green qPCR MasterMix II (SEVEN Biotech, Beijing, China) and gene‐specific primers. The sequences of the CASP10 were CASP10‐F, 5′‐TAGGATTGGTCCCCAACAAGA‐3′ and CASP10‐R, 5′‐GAGAAACCCTTTGTCGGGTGG‐3′. Relative gene expression was calculated by the 2^−ΔΔ*Ct*^ method and normalised to GAPDH.

### 2.12 Immunofluorescence (IF) Analysis

Following the indicated treatments, HUVECs were fixed with 4% paraformaldehyde for 15 min at room temperature, permeabilized with 0.5% Triton X‐100 for 15 min and blocked with 5% BSA for 30 min. Cells were then incubated overnight at 4°C with primary antibodies directed against CASP10 (Abways, Shanghai, China, CY5747, 1:100). After three washes in PBS, species‐matched secondary antibodies conjugated to FITC or ABflo 647 were applied for 1 h at ambient temperature. Nuclei were counterstained with DAPI (Seven Biotechnology, Beijing, China, SI103, undiluted) for 5 min in an anti‐fade mounting medium. Fluorescence images were acquired on an Olympus IX3 microscope (Tokyo, Japan). Quantitative analysis of fluorescence‐positive cells was performed with Image J (National Institutes of Health, USA) using an identical threshold for all images.

### 2.13 Western Blotting

Whole‐cell lysates from HUVECs were prepared in RIPA buffer containing protease and phosphatase inhibitors. Equal amounts of protein were resolved on 10%–15% SDS‐PAGE gels and electro‐transferred to PVDF membranes (Millipore, Billerica, USA). Following that, the PVDF membranes were incubated with primary antibodies specific for CASP10 (Abcam, Waltham, USA, AB177475, 1:5000), NLRP3 (Proteintech, Wuhan, China, 19771‐1‐AP, 1:1000), Caspase‐1 P20 (Immunoway, YT5743, 1:1000), GSDMD and its N‐terminal (Immunoway, Suzhou, China, YT7991, 1:2000), IL‐1β (Proteintech, Wuhan, China, 16806‐1‐AP, 1:4000) and γH_2_AX (Affinity, Jiangsu, China, #AF3187, 1:4000) at 4°C overnight. β‐Actin (Abways, Shanghai, China, AB0035, 1:150000) and β‐Tubulin (Affinity, Jiangsu, China, #AF7011, 1:500000) served as a loading control. After three washes with TBST, membranes were probed with Peroxidase‐Conjugated Goat anti‐Rabbit ihG (H + L) secondary antibodies (ZSGB‐BIO, Beijing, China, ZB‐2301, 1:20000) for 1 h at 37°C. Immunoreactive bands were visualised with an enhanced chemiluminescence substrate and captured using the ChemiDoc XRS imaging system (Bio‐Rad Laboratories, Hercules, CA, USA). Band intensities were quantified by densitometry with Image J (NIH, USA).

### 2.14 Statistical Analysis

Data analysis and visualisation were carried out in R 4.4.3. Group comparisons were evaluated with GraphPad Prism 9 (GraphPad Software, San Diego, CA, USA): two‐group differences were tested by two‐tailed unpaired Student's *t*‐test, whereas multi‐group comparisons used one‐way ANOVA. Gene‐expression comparisons in bioinformatics analyses were performed with the Wilcoxon rank‐sum test. All values are presented as mean ± standard deviations; *p* < 0.05 was considered statistically significant.

## 3 Results

### 3.1 Identification and Functional Enrichment Analysis of DDR‐Associated DEGs

**FIGURE 2:**
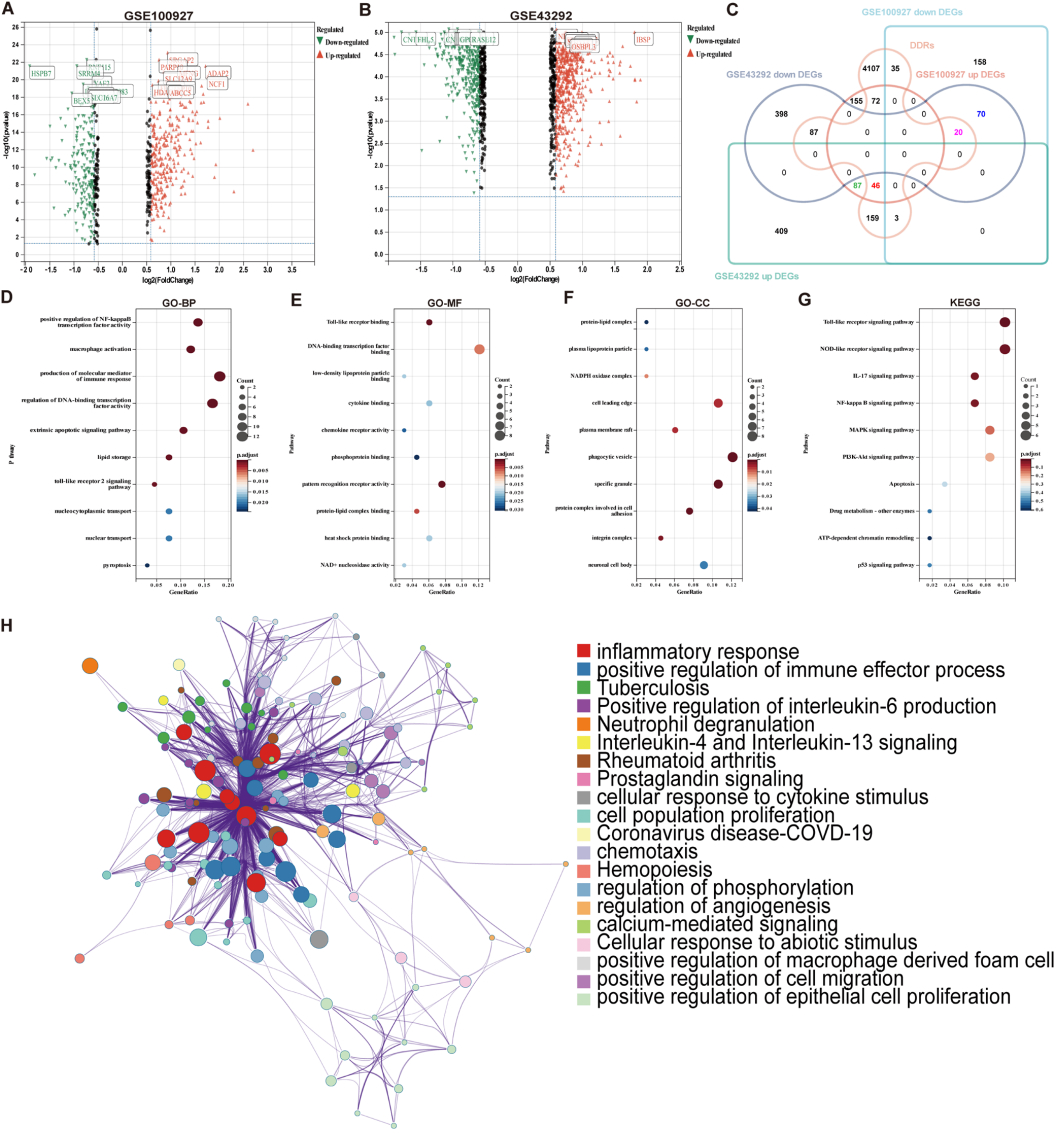
Identification and functional enrichment analysis of DDR‐associated genes in atherosclerosis. (A, B) Volcano plots depicting the distribution of DEGs in the GSE100927 and GSE43292 datasets. (C) A Venn diagram illustrating the common DEGs between the GSE100927 and GSE43292 datasets and their overlap with DDR‐associated genes. The sum of the green and red values represents the up‐regulated DEGs from the two datasets, while the sum of the pink and blue values represents the down‐regulated DEGs. The sum of the pink and red values indicates the DDR‐DEGs that are shared between the DDR and DEG categories. (D–G) GO and KEGG enrichment analysis of DDR‐DEGs performed in R. (H) Comprehensive functional enrichment analysis of DDR‐DEGs using the MetaScape platform, including GO, KEGG, Reactome and WikiPathways analyses.

In the dataset GSE100927, a total of 646 DEGs were identified, comprising 360 upregulated genes and 286 downregulated genes. In parallel, the dataset GSE43292 exhibited 1279 DEGs, consisting of 704 upregulated and 575 downregulated genes (Figure 2A,B). Utilising Venn analysis to intersect the DEGs from these two datasets, we identified 133 upregulated and 90 downregulated genes that are associated with AS. Upon further intersection with gene sets related to DDR, a subset of 66 DDR‐associated DEGs was identified (Figure 2C; for detailed information, refer to File S4). Subsequent GO and KEGG enrichment analyses demonstrated that these genes were significantly enriched in various biological processes, including the production of molecular mediators of immune response, nucleocytoplasmic transport, pyroptosis, DNA‐binding transcription factor binding, pattern recognition receptor activity, protein‐lipid complex, NOD‐like receptor signalling pathway and inflammatory response (Figure 2D–H).

### 3.2 DDR‐Associated DEGs as Differential Markers for Distinguishing AS Plaque Types

**FIGURE 3:**
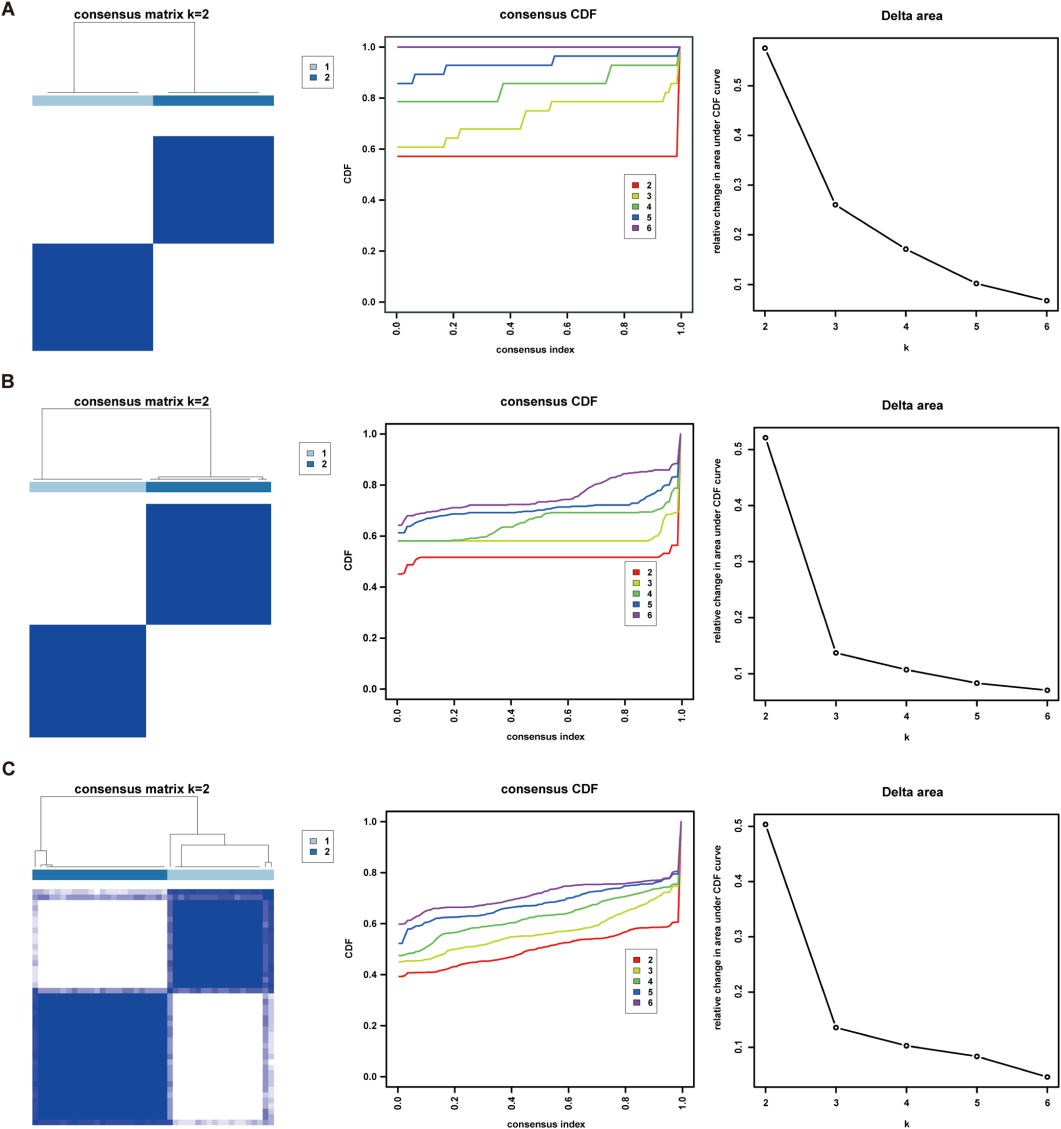
Consensus‐clustering identification of plaque types based on DDR‐related DEGs. (A–C) Results for GSE120521 (unstable vs. stable plaques), GSE128829 (early vs. advanced plaques) and GSE163154 (IPH vs. non‐IPH plaques), respectively. For each dataset, the corresponding panel presents the consensus matrix, cumulative distribution function (CDF) curve and relative change in area under the CDF, which were jointly used to determine the optimal cluster number.

We performed cluster analysis on different types of AS plaques in three datasets: GSE28829, GSE120521 and GSE163154, using the 66 DDR‐associated DEGs. Within the GSE120521 dataset, DDR‐associated DEGs effectively distinguished between stable and unstable plaques (100% vs. 100%) (Figure 3A). In the GSE28829 dataset, early‐stage plaques were predominantly clustered in cluster 1 (92%), while late‐stage plaques were mainly clustered in cluster 2 (81%) (Figure 3B). As depicted in Figure 3C, the majority of AS samples in cluster 1 were characterised as intraplaque haemorrhage (IPH) atherosclerotic (23/27, 85%), and the vast majority of samples in cluster 2 were identified as non‐IPH atherosclerotic (15/16, 94%) in the GSE163154 dataset (Figure 3C). These results demonstrate that DDR‐associated DEGs can accurately differentiate the stages as well as the stability characteristics of AS plaques.

### 3.3 Identification of Key DDR Genes and Construction of a Diagnostic Model of AS

**FIGURE 4:**
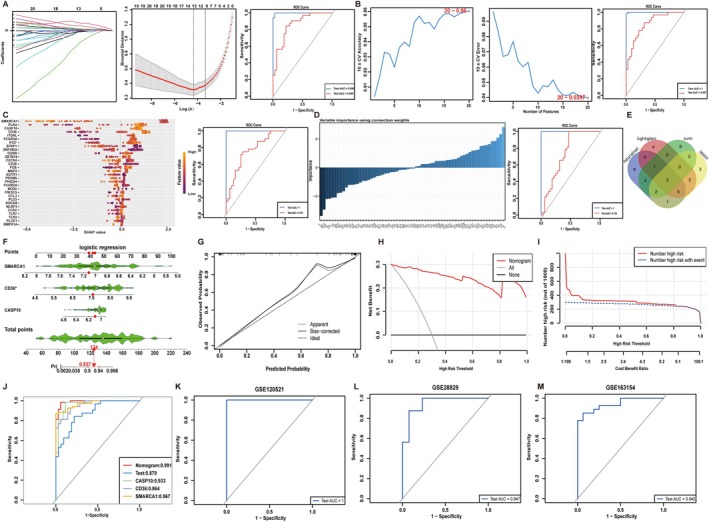
Identify hub genes among DDR‐related DEGs in AS to build a robust diagnostic model. (A) LASSO regression model for feature selection. (B) SVM‐REF model, iteratively removing the least important features to refine the selection of DDR‐associated DEGs. (C) LightGBM algorithm employing SHAP (SHapley Additive exPlanations) values and Neutral network (D) to rank DEGs by feature importance. The area under the ROC curve (AUC) serves as a quantitative metric reflecting the model's capacity for discrimination. (E) Venn diagram depicting the overlap of key diagnostic genes identified by four distinct machine learning algorithms. (F) Nomogram constructed from three key diagnostic genes, designed to predict the progression of AS. (G–I) Calibration curves, Decision curve analysis (DCA) and Clinical impact curves utilised to assess the accuracy of the nomogram in predicting AS progression. (J) Comparative analysis of the receiver operating characteristic (ROC) curves between the nomogram and individual key diagnostic genes, demonstrating the superior predictive power of the integrated nomogram model. (K–M) Evaluation of the diagnostic value of key diagnostic genes in different atherosclerotic statuses across three independent datasets (GSE120521, GSE28829, GSE163154).

Using GSE100927 for training set and GSE43292 for validation set, we benchmarked four machine‐learning frameworks: LASSO (*λ* = 0.0130) retained top 13 predictors (AUC = 0.998/0.859) (Figure 4A), SVM‐RFE identified 20 diagnostic genes (AUC = 1.000/0.857) (Figure 4B), LightGBM‐SHAP ranked top 30 important features, in conjunction with the SHAP algorithm (1.000/0.757) (Figure 4C), and Neural Network analysis was conducted to establish a diagnostic gene importance hierarchy through the comprehensive weight calculation of all genes (1.000/0.738) (Figure 4D). Venn intersection of the ranked gene lists yielded a minimal, robust signature comprising three high‐importance genes: CASP10, CD36 and SMARCA1 (Figure 4E). Based on these genes, we developed a diagnostic nomogram for AS using the GSE100927 dataset. Taking the 11th sample as an example, we individually scored the expression levels of the three key genes, resulting in a cumulative score of 124, which corresponds to an 83.7% predicted risk of developing AS (Figure 4F). Calibration, decision curve analysis (DCA), and clinical impact curves confirmed excellent fit and net benefit (Figure 4G–I). The predictive model built on GSE100927 and validated in GSE43292 achieved a composite AUC of 0.991, surpassing the individual gene performances (Figure 4J). Furthermore, in three independent external cohorts (GSE120521, GSE28829, GSE163154), the three‐gene signature accurately discriminated stable from unstable plaques (AUC = 1.00), early‐stage from advanced plaques (AUC = 0.947) and hemorrhagic from non‐hemorrhagic plaques (AUC = 0.942) (Figure 4K–M).

### 3.4 Nuclear Endothelial CASP10 Drives oxLDL‐Induced DNA Damage in Early AS

**FIGURE 5:**
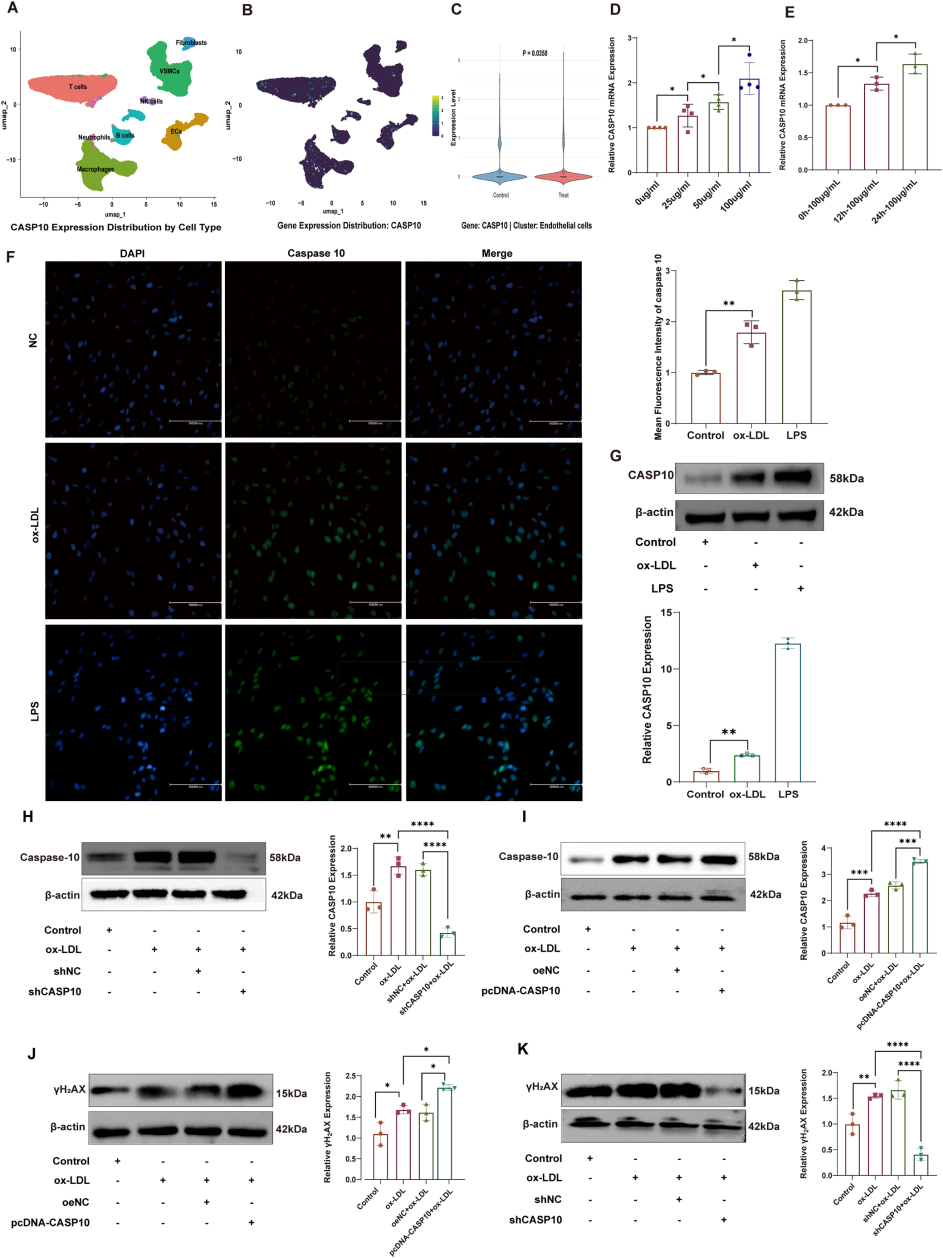
CASP10 expression in AS plaques at single‐cell level and its functional effects on the DDR in ox‐LDL treated HUVECs. (A) UMAP visualisation depicting the clustering results, identifying 8 distinct cell clusters within the AS plaques. (B, C) Differential expression analysis of CASP10 across eight distinct cell types, specifically in ECs, with statistical significance denoted as *p* < 0.05. (D, E) qRT‐PCR analysis of CASP10 mRNA levels in human umbilical vein endothelial cells (HUVECs) treated with various concentrations of ox‐LDL for 24 h and treated with 100 μg/mL ox‐LDL for different durations. (F, G) Fluorescence microscopy images and Western blot (WB) analysis of CASP10 protein in HUVECs treated with 100 μg/mL ox‐LDL for 24 h (*n* = 3). (H, I) Validation of CASP10 knockdown and overexpression efficiency in HUVECs using Western blot analysis (*n* = 3). (J, K) Effects of CASP10 knockdown and overexpression on γH_2_AX expression levels, assessed by Western blot analysis (*n* = 3). *p < 0.05, **p < 0.01, ***p < 0.001, ****p < 0.0001.

Single‐cell RNA‐seq of 35,784 cells from human carotid plaques and adjacent normal arteries (GSE159677) resolved 16 clusters into eight cell types; endothelial cells (ECs) displayed the higher CASP10 expression levels, followed by vascular smooth muscle cells (VSMCs), macrophages, B cells, natural killer (NK) cells, fibroblasts, neutrophils and T cells (Figure 5A–C). To test whether ECs CASP10 participates in the initiation of atherosclerosis, HUVECs were exposed to ox‐LDL. Our findings revealed that ox‐LDL significantly upregulated the expression of CASP10 in a concentration‐ and time‐dependent manner (Figure 5D,E). Based on these results, we selected an ox‐LDL concentration of 100 μg/mL and a 24 h incubation period as the optimal experimental conditions. Western blotting and fluorescence microscopy analyses showed that caspase 10 expression was upregulated in ox‐LDL‐stimulated HUVECs, with caspase 10 predominantly localised in the nucleus (Figure 5F,G). To further explore the functional role of CASP10, we created both CASP10 knockdown and overexpression models in HUVECs (Figure 5H,I). γH_2_AX serves as a highly sensitive marker of DNA damage. Notably, pcDNA‐CASP10 markedly elevated γH_2_AX expression in HUVECs treated with ox‐LDL, compared with the ox‐LDL‐only group (Figure 5J), whereas shRNA targeting CASP10 (shCASP10) suppressed γH_2_AX expression, even when induced by ox‐LDL (Figure 5K). These findings collectively suggest that upregulation of CASP10 potentiates ox‐LDL‐induced DNA damage in endothelial cells, highlighting its role in the early stages of atherosclerosis.

### 3.5 Correlation Analysis of CASP10 Expression With Pyroptosis‐Associated Genes in AS

**FIGURE 6:**
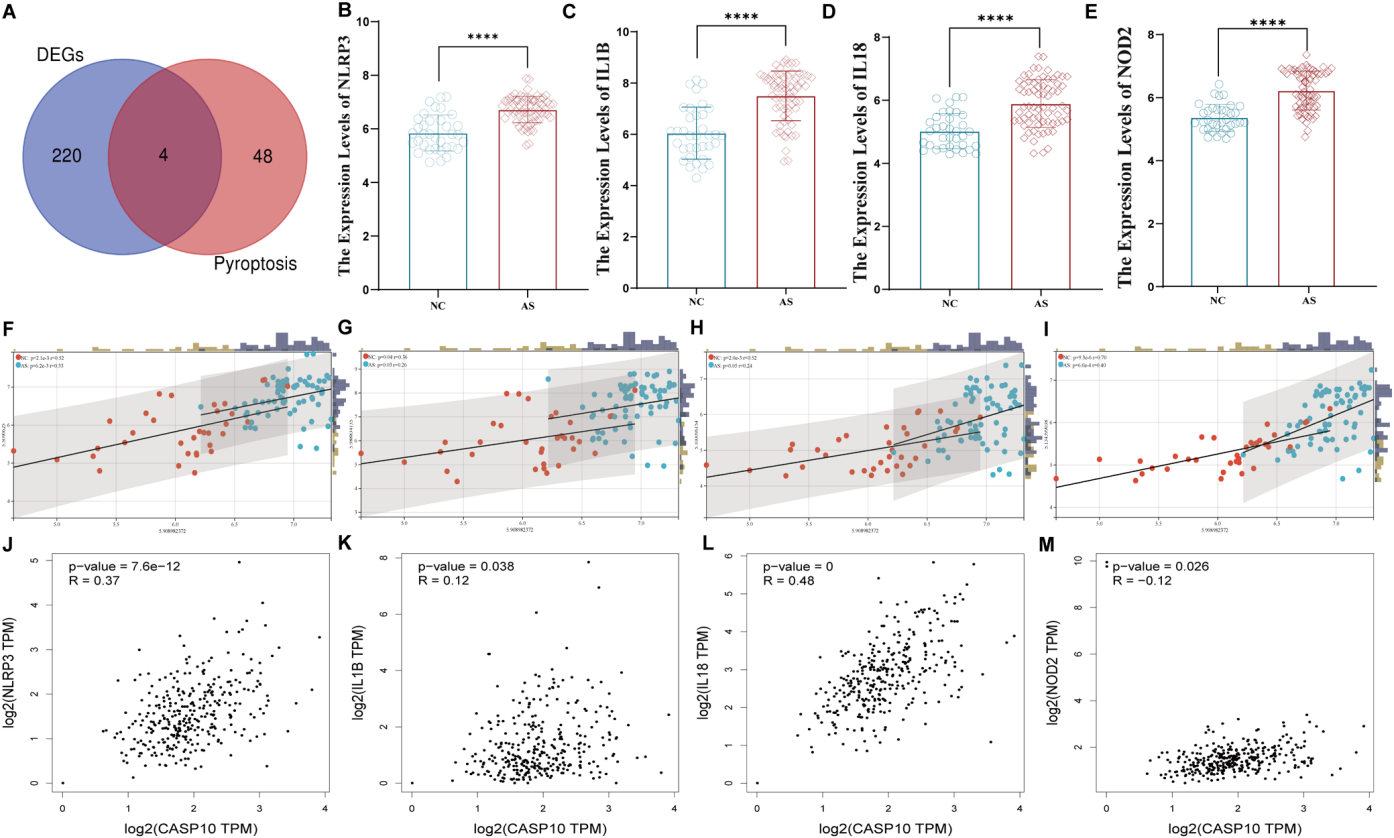
Identification of pyroptosis‐associated DEGs and correlation analysis with CASP10. (A) Venn diagrams illustrating the overlap between DEGs and pyroptosis‐associated genes. (B–E) Boxplot analysis of the expression levels of NLRP3, IL‐1β, IL‐18 and NOD2. (F–I) Correlation analysis between CASP10 and four pyroptosis‐associated DEGs using the sangerbox platform. (J–M) Correlation analysis between CASP10 and four pyroptosis‐associated DEGs using the GEPIA tool. ****p < 0.0001.

Our previous research confirmed the role of ox‐LDL‐induced pyroptosis in HUVECs in AS [21]. Building on this foundation, we conducted a Venn analysis of the 224 atherosclerosis‐related DEGs and the 52 known pyroptosis genes used in our prior studies, identifying four common genes: NLRP3, IL18, IL1β and NOD_2_ (Figure 6A). Compared with normal arterial tissues, the expression levels of these four genes were significantly increased in AS plaques in the GSE100927 dataset (Figure 6B–E). To further elucidate the relationships between these genes and CASP10, we performed co‐expression analysis using both the Sangerbox platform and GEPIA, focusing on aortic and coronary artery tissues. This comprehensive analysis revealed that only NLRP3 and IL1β consistently showed significant positive correlations with CASP10 (*p* < 0.05) (Figure 6F–M).

### 3.6 CASP10 Regulates the HUVECs Pyroptosis Induced by Ox‐LDL

**FIGURE 7:**
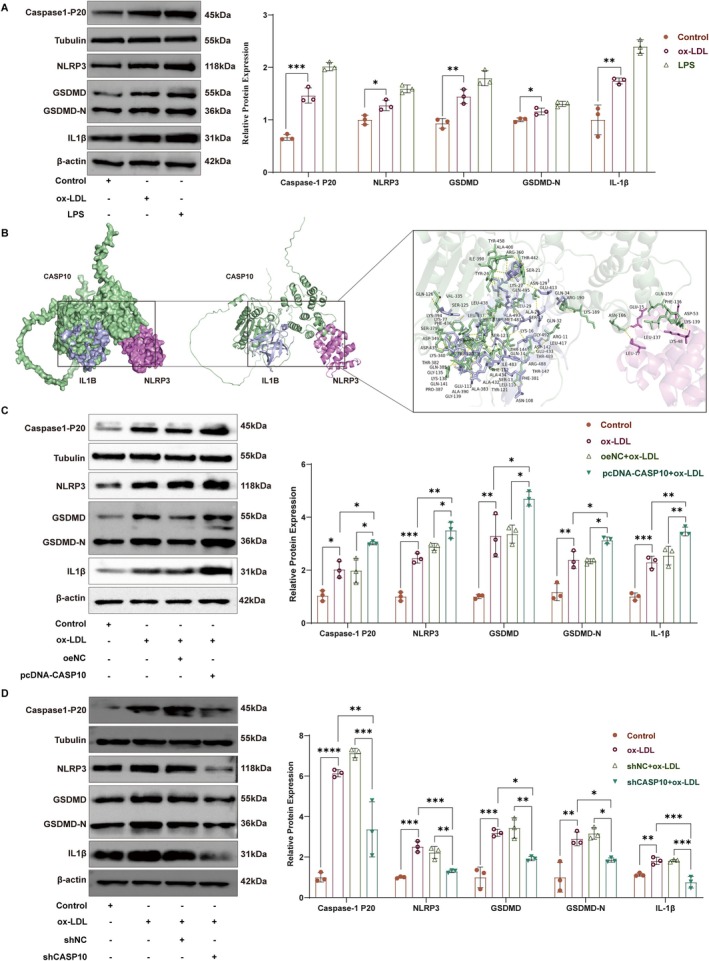
CASP10 regulates the HUVECs pyroptosis. (A) WB analysis of Caspase 1‐P20, NLRP3, GSDMD, GSDMD‐N and IL‐1β protein in HUVECs treated with 100 μg/mL ox‐LDL for 24 h (*n* = 3). (B) Molecular docking analysis identifies direct binding sites of CASP10 with IL‐1B and NLRP3. (C) Overexpression of CASP10 in HUVECs treated with 100 μg/mL ox‐LDL for 24 h modulates the expression levels of NLRP3, Caspase 1‐P20, GSDMD, GSDMD‐N and IL‐1β (*n* = 3). (D) Knockdown of CASP10 in HUVECs treated with 100 μg/mL ox‐LDL for 24 h modulates the expression levels of NLRP3, Caspase 1‐P20, GSDMD, GSDMD‐N and IL‐1β (*n* = 3). β‐Actin served as the loading control for NLRP3, GSDMD, GSDMD‐N and IL‐1β blots; β‐tubulin was used for Caspase‐1 p20 blots. *p < 0.05, **p < 0.01, ***p < 0.001, ****p < 0.0001.

We first confirmed the expression of pyroptosis‐related molecules in HUVECs. Consistent with our previous studies, NLRP3 and IL1β were upregulated in HUVECs treated with ox‐LDL. Additionally, the expressions of GSDMD‐N and caspase 1‐P20 were also increased (Figure 7A). Further, molecular docking simulations revealed that CASP10 specifically interacts with both NLRP3 and IL1B in a spatially defined manner (Figure 7B). Specifically, residues 136–166 of CASP10 form a critical motif essential for NLRP3 binding, whereas residues 189–190 and 330–495 likely represent key structural determinants for IL‐1β targeting. pcDNA‐CASP10 significantly elevated the expression levels of pyroptosis‐related molecules in HUVECs that were previously induced by ox‐LDL (Figure 7C). Conversely, shCASP10 markedly reduced the expression levels of pyroptosis‐related molecules in HUVECs, even under ox‐LDL induction (Figure 7D). Collectively, these findings indicate that upregulation of CASP10 potentiates ox‐LDL‐induced pyroptosis in endothelial cells, whereas CASP10 knockdown significantly mitigates this effect.

## 4 Discussion

Atherosclerosis is now widely recognised as a disease propelled by chronic endothelial injury and maladaptive inflammatory responses. Our study leverages multi‐transcriptomic data, machine learning biomarker discovery and stringent functional validation to identify CASP10 as a previously underexplored DDR‐related biomarker that links genotoxic stress to endothelial pyroptosis. These findings extend the current understanding of atherosclerosis pathogenesis by situating CASP10 at the nexus of vascular DDR and inflammasome activation.

We initially performed an integrated analysis of three independent transcriptome datasets, identifying 66 differentially expressed genes associated with the DDR. The transcriptional signatures of these genes effectively distinguished stable from unstable plaques, early from advanced lesions and hemorrhagic from non‐hemorrhagic plaques. This cross‐cohort consistency highlights the broad applicability of our gene set and supports its potential as a molecular classifier for plaque vulnerability. Notably, unsupervised clustering achieved over 80% accuracy across three external datasets, with perfect accuracy (100%) in differentiating plaque stability. While the modest sample size of this cohort may influence these results, DDR‐related genes demonstrated remarkable performance in risk stratification for characterising atherosclerotic plaques, suggesting that DDR signatures could enhance current risk‐stratification algorithms.

To further identify the key DDR‐related genes associated with AS, we employed a comprehensive approach combining LASSO, SVM‐RFE, LightGBM‐SHAP and neural network analyses, ultimately pinpointing three highly reliable diagnostic genes: CASP10, CD36 and SMARCA1. The combination of these three genes demonstrated exceptional diagnostic performance for AS (AUC = 0.991), outperforming the previously identified 66 DDR‐related genes in distinguishing the three types of AS plaques (AUC > 0.94). There is a wealth of research on the association between CD36 and AS. CD36 is involved in regulating inflammation, lipid metabolism and the functions of endothelial cells and smooth muscle cells [22, 23, 24]. In atherosclerotic lesions, CD36 is localised in the intimal regions that are rich in macrophages and is associated with plaque instability [25, 26]. Targeting the CD36 protein can identify vulnerable atherosclerotic plaques [27]. However, through the ROC curve analysis of the validation set, we found that the diagnostic value of CD36 was relatively low, with the area under the AUC curve being only 0.864. Despite SMARCA1 having the highest diagnostic value, our experimental validation revealed a discrepancy between the expression of SMARCA1 in HUVECs induced by ox‐LDL and the corresponding transcriptomic data of AS (File S5). Given its diagnostic efficacy, unique role in endothelial genotoxic responses, and consistent transcriptional and translational profiles in ox‐LDL‐stimulated HUVECs, CASP10 was prioritised for further investigation.

CASP10, a member of the caspase family located at human chromosome locus 2q33‐34, shares high homology with CASP8 [28]. It is essential for cell survival, promoting NF‐κB activation via interactions with RIP, NIK and IKKα, and participating in non‐apoptotic or anti‐apoptotic signalling pathways [29, 30]. In recent years, the role of CASP10 in tumours has been extensively reported. Elevated CASP10 expression correlates positively with tumour grade in renal clear cell carcinoma and is associated with unfavourable overall survival in gastric cancer patients [28, 31]. Additionally, CASP10 upregulation can drive the progression of dilated cardiomyopathy by inducing cardiomyocyte apoptosis [32]. These studies highlight the involvement of CASP10 in various diseases, yet its role in the pathogenesis of atherosclerosis remains unexplored. Our single‐cell analysis of carotid artery plaques confirmed elevated CASP10 expression in endothelial cells of AS plaques, aligning with transcriptome data and providing spatial resolution. This selective enrichment in endothelial cells may reflect their position as the first vascular layer exposed to circulating ox‐LDL, making them primary sensors of genotoxic stress. Functional experiments in HUVECs demonstrated that CASP10 is both necessary and sufficient for ox‐LDL‐induced DNA damage and pyroptosis of HUVECs.

Certain viral infections or ultraviolet damage can lead to the disruption of nuclear membrane integrity during the disease process, resulting in the release of double‐stranded DNA into the cytoplasm and subsequent activation of the NLRP3 [33, 34]. However, AS is a non‐infectious low‐grade inflammatory disease, suggesting the potential for distinct mechanisms of NLRP3 inflammasome activation. Activation of the NLRP3 inflammasome culminates in the expression and secretion of mature IL‐1β into the extracellular milieu, epitomising the canonical pyroptotic pathway [35]. The abnormal activation of NLRP3 inflammasomes is implicated in atherosclerosis progression in both mice and humans [36, 37]. Cholesterol crystals activate the NLRP3 inflammasome, leading to the production of pro‐inflammatory cytokines IL‐1β and IL‐1α in endothelial cells [38]. This activation links cholesterol to sterile inflammation, a key feature of atherosclerosis. Elevated levels of circulating IL‐1β and IL‐18 are associated with increased macrophage recruitment, accelerated foam cell formation and plaque progression [39]. Studies in ApoE‐deficient mice and humans have shown that IL‐1β inhibition reduces atherosclerosis risk, highlighting IL‐1β as a central driver of lesion progression [40]. However, neutralising or inhibiting exogenous IL‐1β does not address its ongoing production. IL‐1β can still bind to cholesterol transport proteins, impairing cholesterol efflux and sustaining foam cell formation. Therefore, directly targeting IL‐1β production by the NLRP3 inflammasome may offer a more effective therapeutic strategy than merely neutralising or inhibiting exogenous IL‐1β, as it addresses the continuous production of this cytokine [41]. Our previous experiments have confirmed that interventions targeting endothelial pyroptosis can reduce endothelial NLRP3 and IL‐1β expression, thereby effectively alleviating atherosclerotic plaque progression [18]. Given that our enrichment analyses specifically linked DDR‐related features to pyroptosis, and identified NOD‐like receptor signalling and inflammatory cascade pathways as associated nodes, we hypothesized that CASP10 might serve as a nexus between DNA damage perception and inflammasome activation. Consistent with this hypothesis, overexpression of CASP10 exacerbated γH_2_AX accumulation, increased NLRP3 expression, GSDMD‐N cleavage and IL‐1β release. Conversely, CASP10 knockdown reversed these effects. These findings establish CASP10 as a direct sensor‐transducer of endothelial DNA damage, acting upstream of NLRP3 and highlighting its regulatory role in ox‐LDL‐stimulated endothelial pyroptosis. This suggests that CASP10 may represent an early and novel target for modulating AS plaque progression, particularly for stabilising plaques that are prone to becoming unstable.

Despite the excellent calibration and net clinical benefit of CASP10 demonstrated in decision curve analysis, which suggests its potential to identify patients at high risk for plaque rupture who may benefit from intensified anti‐inflammatory or DNA damage‐targeted therapies, several limitations must be acknowledged. First, our transcriptomic data were derived from carotid endarterectomy samples, which may not fully reflect the situation in coronary atherosclerosis. Second, although we verified the function of CASP10 in primary endothelial cells, the absence of CASP10 in mice precluded in vivo rescue experiments targeting endothelial cell‐specific CASP10 deletion. This limitation necessitates further research to develop strategies that can provide in vivo evidence demonstrating that downregulation of CASP10 alleviates AS and promotes plaque stability. Third, although we have provided evidence of the molecular docking sites between CASP10 and NLRP3, the precise molecular interface between CASP10 and the NLRP3 inflammasome, whether direct or mediated via the cyclic GMP‐AMP synthase‐stimulator of interferon genes (cGAS‐STING) pathway or absent in melanoma 2 (AIM2), still requires further deconstruction and interaction studies [33].

In conclusion, our study highlights the effectiveness of a DDR‐centered multi‐omics strategy in identifying clinically relevant targets within the atherosclerosis landscape. CASP10 emerges as a dual entity, serving as a reliable biomarker for identifying unstable plaques and functioning as a pivotal endothelial mediator that links DNA damage to pyroptotic inflammatory cascades. Accordingly, targeting CASP10 via pharmacological or genetic means may represent a novel therapeutic strategy aimed at reducing endothelial cell death and enhancing atherosclerotic plaque stability.

## Author Contributions

**Wenjing Zhang:** writing – review and editing, supervision, funding acquisition. **Xiangrong Meng:** writing – original draft, formal analysis, methodology, visualization, conceptualization. **Xinyu Zhu:** methodology. **Yu Xi:** data curation. **Zhuozhong Wang:** project administration. **Kejian Zhang:** data curation, investigation.

## Funding

This work was supported by National Natural Science Foundation of China (Grant Nos. 82001715, 82204147), Research Project of the Health and Family Planning Commission of Heilongjiang Provincial (Grant No. 2018286), Fundamental Research Funds for the Provincial Universities of Heilongjiang (Grant No. 2017LCZX67).

## Ethics Statement

The present study was approved by the Ethics Committee of the Second Affiliated Hospital of Harbin Medical University (Approval No. KY2022‐312).

## Consent

Written informed consent was obtained from all participants.

## Conflicts of Interest

The authors declare no conflicts of interest.

## Supporting information


**File S1:** Basic information of GEO datasets used in the study.

**File S2:** Cell annotation markers [42, 43, 44].

**File S3:** Target sequences of CASP10.

**File S4:** DDR‐associated DEGs.

**File S5:** Relative SMARCA1 mRNA Expression.

## Data Availability

The raw and processed transcriptomic data that support the findings of this study have been deposited in NCBI's Gene Expression Omnibus and are accessible through GEO Series accession number GSE100927, GSE43292, GSE28829, GSE120521, GSE163154 and GSE159677 at https://www.ncbi.nlm.nih.gov/geo/.
