# Supplementary material for: Systematic Identification and Functional Validation of CASP10 as a DNA‐Damage‐Responsive Driver of Endothelial Pyroptosis in Atherosclerosis
Source: J Cell Mol Med. 2026 Feb 17;30(4):e71060. doi: 10.1111/jcmm.71060 (PMC12912935; doi:10.1111/jcmm.71060)
Supplement: Supplementary file 3 — File S3: Target sequences of CASP10. [file JCMM-30-e71060-s003.docx]

**Target sequences of CASP10**

The target sequence of CASP10

ATGAAATCTCAAGGTCAACATTGGTATTCCAGTTCAGATAAAAACTGTAAAGTGAGCTTTCGTGAGAAGCTTCTGATTATTGATTCAAACCTGGGGGTCCAAGATGTGGAGAACCTCAAGTTTCTCTGCATAGGATTGGTCCCCAACAAGAAGCTGGAGAAGTCCAGCTCAGCCTCAGATGTTTTTGAACATCTCTTGGCAGAGGATCTGCTGAGTGAGGAAGACCCTTTCTTCCTGGCAGAACTCCTCTATATCATACGGCAGAAGAAGCTGCTGCAGCACCTCAACTGTACCAAAGAGGAAGTGGAGCGACTGCTGCCCACCCGACAAAGGGTTTCTCTGTTTAGAAACCTGCTCTACGAACTGTCAGAAGGCATTGACTCAGAGAACTTAAAGGACATGATCTTCCTTCTGAAAGACTCGCTTCCCAAAACTGAAATGACCTCCCTAAGTTTCCTGGCATTTCTAGAGAAACAAGGTAAAATAGATGAAGATAATCTGACATGCCTGGAGGACCTCTGCAAAACAGTTGTACCTAAACTTTTGAGAAACATAGAGAAATACAAAAGAGAGAAAGCTATCCAGATAGTGACACCTCCTGTAGACAAGGAAGCCGAGTCGTATCAAGGAGAGGAAGAACTAGTTTCCCAAACAGATGTTAAGACATTCTTGGAAGCCTTACCGCAGGAGTCCTGGCAAAATAAGCATGCAGGTAGTAATGGTAACAGAGCCACAAATGGTGCACCAAGCCTGGTCTCCAGGGGGATGCAAGGAGCATCTGCTAACACTCTAAACTCTGAAACCAGCACAAAGAGGGCAGCTGTGTACAGGATGAATCGGAACCACAGAGGCCTCTGTGTCATTGTCAACAACCACAGCTTTACCTCCCTGAAGGACAGACAAGGAACCCATAAAGATGCTGAGATCCTGAGTCATGTGTTCCAGTGGCTTGGGTTCACAGTGCATATACACAATAATGTGACGAAAGTGGAAATGGAGATGGTCCTGCAGAAGCAGAAGTGCAATCCAGCCCATGCCGACGGGGACTGCTTCGTGTTCTGTATTCTGACCCATGGGAGATTTGGAGCTGTCTACTCTTCGGATGAGGCCCTCATTCCCATTCGGGAGATCATGTCTCACTTCACAGCCCTGCAGTGCCCTAGACTGGCTGAAAAACCTAAACTCTTTTTCATCCAGGCCTGCCAAGGTGAAGAGATACAGCCTTCCGTATCCATCGAAGCAGATGCTCTGAACCCTGAGCAGGCACCCACTTCCCTGCAGGACAGTATTCCTGCCGAGGCTGACTTCCTACTTGGTCTGGCCACTGTCCCAGGCTATGTATCCTTTCGGCATGTGGAGGAAGGCAGCTGGTATATTCAGTCTCTGTGTAATCATCTGAAGAAATTGGTCCCAAGACATGAAGACATCTTATCCATCCTCACTGCTGTCAACGATGATGTGAGTCGAAGAGTGGACAAACAGGGAACAAAGAAACAGATGCCCCAGCCTGCTTTCACACTAAGGAAAAAACTAGTATTCCCTGTGCCCCTGGATGCACTTTCATTATAG

Note: This file corresponds to Supplementary file S3 as cited.
