# Supplementary material for: Systematic Identification and Functional Validation of CASP10 as a DNA‐Damage‐Responsive Driver of Endothelial Pyroptosis in Atherosclerosis
Source: J Cell Mol Med. 2026 Feb 17;30(4):e71060. doi: 10.1111/jcmm.71060 (PMC12912935; doi:10.1111/jcmm.71060)
Supplement: Supplementary file 4 — File S4: DDR‐associated DEGs. [file JCMM-30-e71060-s005.docx]

**DDR-associated DEGs**

CXCR4

FGF1

IFI27

BTK

TFEC

PHGDH

IL18

ITGB2

MME

MMP1

FCGR2A

F13A1

ERBB2

CD28

PTPN6

PRDM5

LPL

MAF

ITGAL

HSPB8

BMPR1A

RAC2

DOCK8

CD36

PRKCD

ZBTB16

ITGAM

PIK3CG

SYK

FOS

MAP2

PYGM

AR

SFRP1

NPC1

PLCE1

TLR1

PRDM16

SMAD9

FCGR3A

MSR1

SMARCA1

PTPRC

MYLK

TLR7

CLEC7A

CYBB

GUSB

TPM2

IDH1

IL1B

NOD2

TLR2

PLAU

ALOX5

NLRP3

PKD2

CASP10

CCR2

AGTR1

PLD3

YAP1

PTGS1

ZNF385A

CCL3

MMP9

Note: This file corresponds to Supplementary file S4 as cited.
