# Supplementary figures and images for: Systematic Identification and Functional Validation of CASP10 as a DNA‐Damage‐Responsive Driver of Endothelial Pyroptosis in Atherosclerosis
Source: J Cell Mol Med. 2026 Feb 17;30(4):e71060. doi: 10.1111/jcmm.71060 (PMC12912935; doi:10.1111/jcmm.71060)

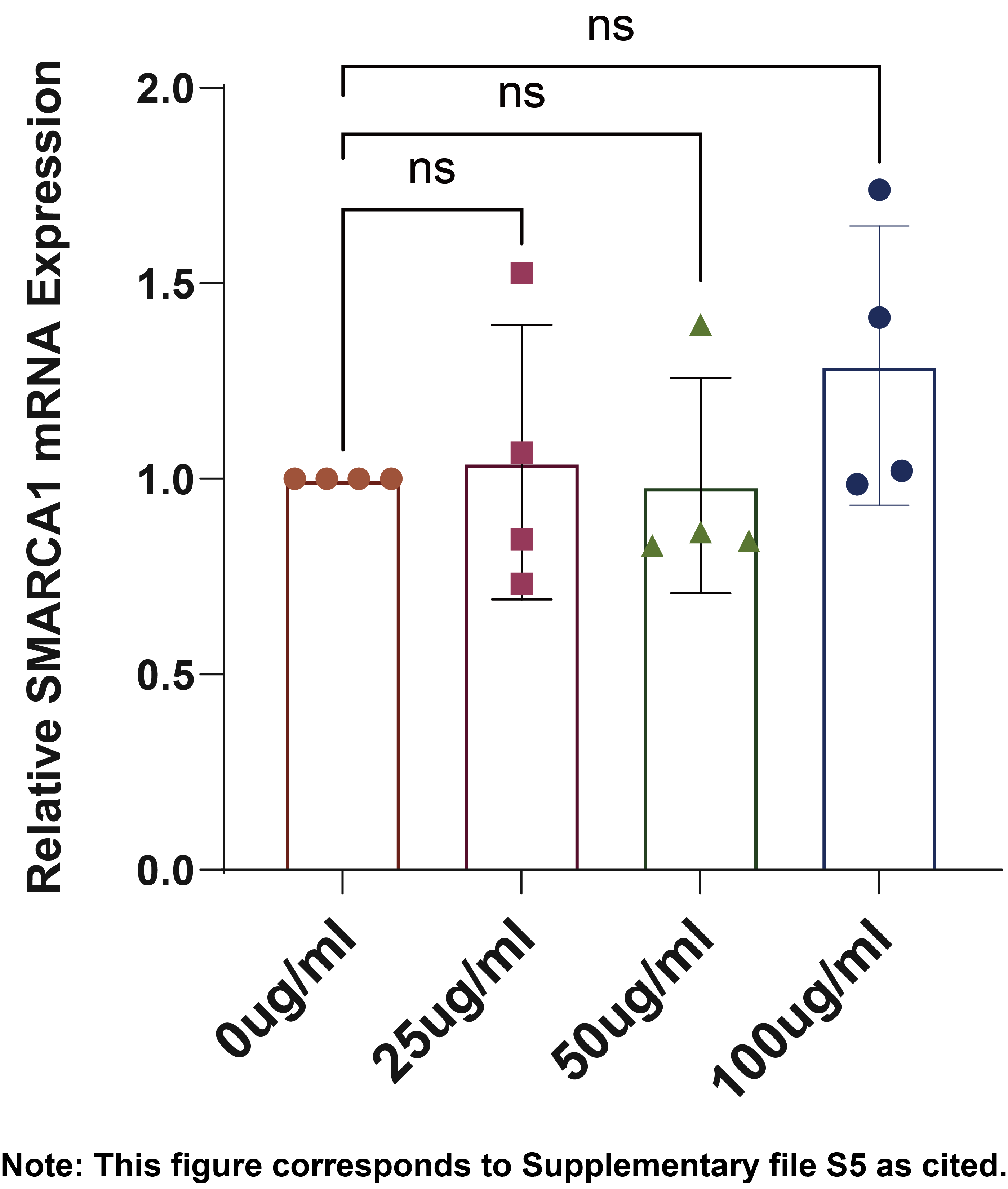

Supplement: Supplementary file 5 — File S5: Relative SMARCA1 mRNA Expression. [file JCMM-30-e71060-s004.png]
